# Natural Honey-Propolis Combinations with Health-Promoting Potential: Antibacterial Activity Against Foodborne Pathogens

**DOI:** 10.3390/ph18070988

**Published:** 2025-07-01

**Authors:** Vanesa Sánchez-Martín, Marta B. López-Parra, Amaia Iriondo-DeHond, Aneta Wojdyło, Anna Michalska-Ciechanowska, Ana I. Haza, Paloma Morales, María Dolores del Castillo

**Affiliations:** 1 Instituto de Investigación en Ciencias de la Alimentación, Consejo Superior de Investigaciones Científicas (CSIC)-Universidad Autónoma de Madrid (UAM), 28049 Madrid, Spain; vanesa.s@csic.es (V.S.-M.); martab.lopez.parra@csic.es (M.B.L.-P.); 2 Departamento de Nutrición y Ciencia de los Alimentos, Sección Departamental de Nutrición y Ciencia de los Alimentos, Facultad de Veterinaria, Universidad Complutense, 28040 Madrid, Spain; amaiairi@ucm.es (A.I.-D.); hanais@ucm.es (A.I.H.); pmorales@ucm.es (P.M.); 3 Department of Fruit, Vegetable and Plant Nutraceutical Technology, The Faculty of Biotechnology and Food Science, Wrocław University of Environmental and Life Sciences, Chełmońskiego 37, 51-630 Wrocław, Poland; aneta.wojdylo@upwr.edu.pl (A.W.); anna.michalska@upwr.edu.pl (A.M.-C.)

**Keywords:** *Clostridium perfringens*, *Listeria monocytogenes*, foodborne pathogens, honey-propolis combinations, phenolic compounds

## Abstract

**Background/Objectives:** Natural products such as honey and propolis have been widely studied for their antimicrobial properties. Combining these substances has shown synergistic effects against foodborne pathogens and has also demonstrated promising results in previous applications on fermented meat products. This study evaluated the antibacterial potential of Spanish thyme (*Thymus* spp.) and chestnut (*Castanea sativa*) honeys, enriched with 10% ethanolic extract of propolis, against two major foodborne pathogens: *Listeria monocytogenes* and *Clostridium perfringens*. **Methods:** Antibacterial activity was assessed using broth microdilution assays and colony-forming unit (CFU) counts. The phenolic composition of the most active samples was characterized by LC-MS-Q/TOF and UPLC-PDA to identify and quantify the bioactive compounds. **Results:** All samples exhibited differential responses depending on the pathogen, with *C. perfringens* being the most susceptible. Propolis addition significantly enhanced the bactericidal response of honey against *L. monocytogenes* and *C. perfringens* (*p* < 0.05). This effect correlated with higher levels of antimicrobial phenolic compounds, particularly cinnamic acid derivatives, pinobanksin-3-*O*-hexanoside, sakuranetin, quercetin, and quercetin-3,7-dimethyl ether. **Conclusions:** These findings support the synergistic antibacterial potential of honey-propolis combinations, highlighting their application as natural preservatives for reducing the risk of foodborne diseases, as well as bioactive ingredients in nutraceutical formulations with antibacterial properties and additional health benefits.

## 1. Introduction

Honey and propolis have been widely used in traditional medicine due to their antimicrobial effects, among other health benefits. In recent years, their application in modern pharmacology has gained attention as a potential alternative to conventional antibiotics, especially in light of rising antimicrobial resistance [1]. Numerous studies suggest that phenolic compounds and other bioactive constituents contribute significantly to their pharmacological and nutraceutical potential, positioning them as promising candidates for therapeutic applications beyond food safety [2].

Foodborne diseases remain a major global health concern, causing significant morbidity and mortality. According to the World Health Organization (WHO), approximately one in ten people suffers from foodborne illness each year, resulting in over 420,000 deaths globally [3]. More than 200 different foodborne pathogens have been identified, with clinical manifestations ranging from mild gastrointestinal distress to severe outcomes such as kidney failure, neurological complications, inflammatory syndromes, and even cancer [4].

Among the most relevant foodborne pathogens are *Listeria monocytogenes* (*L. monocytogenes*), and *Clostridium perfringens* (*C. perfringens*) [5]. *L. monocytogenes* is particularly dangerous due to its high fatality rate and ability to survive at refrigeration temperatures, contaminating ready-to-eat foods like soft cheeses and cold cuts [6]. *C. perfringens* is a spore-forming bacterium widely present in the environment and often associated with improperly stored cooked foods [7]. In the European Union, *L. monocytogenes* and *C. perfringens* are among the most frequently reported foodborne pathogens associated with outbreaks and hospitalizations [8]. In Spain, *L. monocytogenes* is a significant public health concern due to its high morbidity and mortality rates. Notably, listeriosis was designated a mandatory notifiable disease by the Spanish Ministry of Health in 2015 [9]. According to the most recent One Health report by the European Food Safety Authority (EFSA) and European Centre for Disease Prevention and Control (ECDC), two of the largest strong-evidence foodborne outbreaks reported in Spain in 2022, affecting 426 and 165 individuals, respectively, were attributed to the consumption of meat and meat products contaminated with *Clostridium perfringens* toxins [10]. Yet, recent analyses suggest that its incidence and fatality rates remain worryingly high, underscoring the need for effective preventive strategies. Studying these pathogens is essential for improving food safety strategies and protecting public health.

The antimicrobial activity of honey is primarily attributed to its osmotic effect, acidity, hydrogen peroxide content, and phenolic profile. Spanish honey, especially those derived from thyme (*Thymus vulgaris* L.) and chestnut (*Castanea sativa* Mill.), is particularly rich in polyphenols and has been shown to exhibit strong antioxidant and antibacterial properties against *Staphylococcus aureus* (*S. aureus*) and *Escherichia coli* (*E. coli*) [11,12]. Combining honey with other bee products like propolis may further enhance its efficacy. Propolis contains high concentrations of phenolic compounds such as pinocembrin, quercetin, chrysin, and artepillin C [13,14] and exhibits antimicrobial activity against pathogens including *L. monocytogenes* and *C. perfringens* [14,15]. Various studies have explored the synergistic antimicrobial effects of honey and propolis against foodborne pathogens. Although synergistic effects were observed against *E. coli* and *S. aureus*, no synergism was detected against *L. monocytogenes* [16,17]. Their composition and bioactivity are highly dependent on their geographic and botanical origins [13].

Recent studies highlight the growing interest in the synergistic antimicrobial action of honey and propolis. For example, Stefanis et al. conducted a comprehensive bibliometric analysis, revealing honey’s co-use with propolis as an emerging trend in food safety and health-related applications [18]. In parallel, advances in analytical techniques—such as the UHPLC-Q-TOF-MS workflow used by Koulis et al. for comprehensive phenolic profiling of honey [19] and the LC-ESI-MS/MS method applied by Cagri Akman et al. to characterize bioactive compounds in propolis extracts [20]—inspired our combined use of UPLC-PDA for quantification and LC-Q/TOF-MS for structural confirmation in this study.

Previous work by our group demonstrated the feasibility of using chestnut honey-propolis mixtures in nitrate-reduced fermented meat products, primarily for their antioxidant function [21]. In that study, the addition of these mixtures preserved the sensory profile and supported fermentation, and the absence of pathogens was observed at the end of ripening. However, the specific contribution of these combinations to antimicrobial activity had not been experimentally confirmed. The present study addresses this gap by characterizing the antibacterial effects of different honey-propolis mixtures under controlled conditions and linking them to their compositional profiles. This research work investigates the antibacterial effect of Spanish thyme and chestnut honeys, both alone and enriched with 10% propolis extract, against *L. monocytogenes* and *C. perfringens*. While the individual antimicrobial properties of these honeys have been previously reported, their combined application with propolis remains largely unexplored. Furthermore, the phenolic composition of the most active samples was characterized by liquid chromatography–mass spectrometry–quadrupole time of flight (LC-MS-Q/TOF) and ultra-performance liquid chromatography coupled to photodiode array detection (UPLC-PDA) to identify and quantify the bioactive compounds responsible for the observed activity. The study supports the potential of honey-propolis combinations in food safety and nutraceuticals.

## 2. Results

### 2.1. Antimicrobial Activity of Honey-Propolis Mixtures

**Figure 1:**
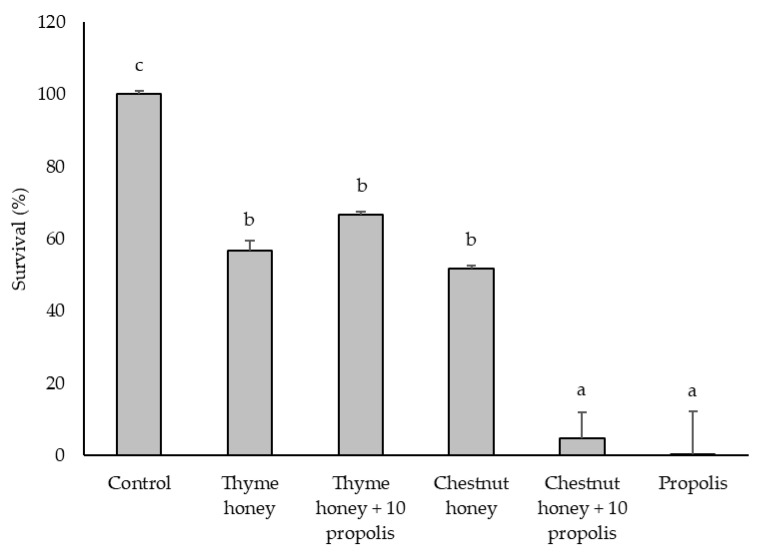
Inhibitory effects of bee product samples (250 mg/mL) on the growth of *L. monocytogenes*. Different letters above the bars indicate statistically significant differences (*p* < 0.05; Tukey’s HSD test). Values represent the means of two independent biological replicates, each consisting of two technical replicates (*n* = 2). Error bars indicate the standard deviation.

**Figure 2:**
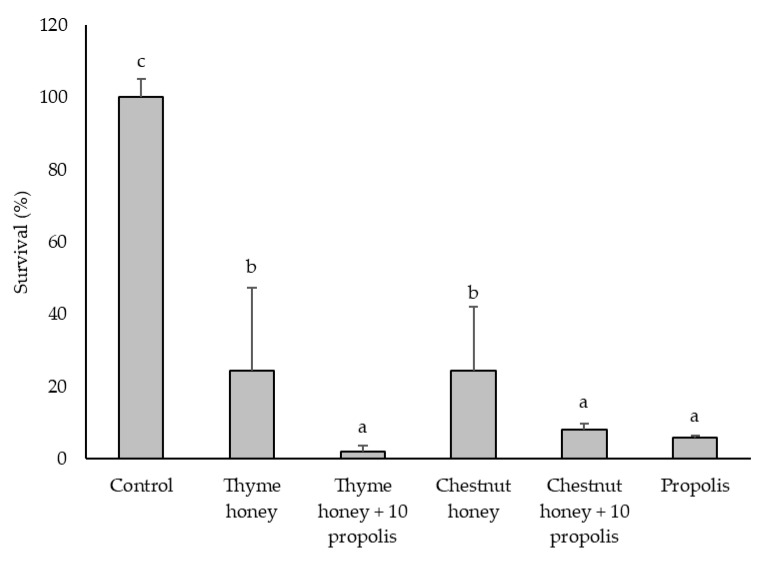
Inhibitory effects of bee product samples (250 mg/mL) on the growth of *C. perfringens*. Different letters above the bars indicate statistically significant differences (*p* < 0.05; Tukey’s HSD test). Values represent the means of two independent biological replicates, each consisting of two technical replicates (*n* = 2). Error bars indicate the standard deviation.

Thyme honey, chestnut honey, propolis, and their combinations exhibited antibacterial activity against *L. monocytogenes* and *C. perfringens*, as assessed by broth microdilution assay. Pathogen survival was evaluated by measuring absorbance at 600 nm (Figure 1 and Figure 2) and confirmed by colony-forming unit (CFU) counts (Table 1).

Both honey types and propolis, as well as thyme honey supplemented with 10% propolis, significantly reduced the survival of *L. monocytogenes* at concentrations starting from 125 mg/mL. Propolis was the most effective treatment, achieving complete inhibition (0% survival) at this concentration. In contrast, chestnut honey with 10% propolis was effective from 250 mg/mL. The antimicrobial effects observed were dose dependent.

Figure 1 shows the percentage survival of *L. monocytogenes* at 250 mg/mL for each sample tested. All treatments reduced bacterial survival, with propolis alone and its combination with chestnut honey displaying the strongest antimicrobial activity. The addition of 10% propolis to chestnut honey significantly enhanced its effect compared to chestnut honey alone, lowering the survival rate to 4.92%. However, this synergistic effect was not observed when propolis was added to thyme honey at the same concentration, suggesting that the enhancement of antibacterial activity by propolis may depend on the honey type. Notably, at 500 mg/mL, a synergistic effect was observed for both combinations: thyme honey with propolis completely inhibited bacterial survival (0%), while chestnut honey with propolis reduced it to 2.92%.

In the case of *C. perfringens*, all samples significantly reduced bacterial survival from 62.5 mg/mL onward, with chestnut honey enriched with 10% propolis and propolis alone showing the strongest antimicrobial effects at this concentration. *C. perfringens* was more susceptible than *L. monocytogenes* to treatment with bee products and their combinations.

The inhibitory effect of the samples at 250 mg/mL on *C. perfringens* growth is shown in Figure 2. The addition of propolis to both honeys significantly enhanced their antibacterial activity. However, the honey-propolis mixtures did not show significantly greater effects compared to propolis alone, underscoring the dominant role of propolis in the observed inhibition. As with *L. monocytogenes*, no significant differences were found between thyme and chestnut honeys when used alone. At 500 mg/mL, both propolis and chestnut honey with 10% propolis completely inhibited *C. perfringens* survival, reducing it to 0%.

The colony-forming unit (CFU) counts (Table 1) obtained 24 h after the absorbance measurements (Figure 1 and Figure 2) provided additional insights, particularly for the propolis and propolis-enriched honey samples. For *L. monocytogenes*, all three samples—propolis, thyme honey + 10% propolis, and chestnut honey + 10% propolis—exhibited a growth-inhibitory effect at 125 mg/mL. In contrast, *C. perfringens* showed growth inhibition at much lower concentrations: from 15.6 mg/mL for propolis and from 31.2 mg/mL for both thyme and chestnut honeys enriched with 10% propolis. These results confirm that *C. perfringens* is more sensitive to the tested samples than *L. monocytogenes* and reinforce the ability of propolis to enhance the antimicrobial activity of honey, even at relatively low concentrations.

The values corresponding to samples with a bactericidal effect are presented in Table 2. Colony-forming unit (CFU) quantification at these concentrations showed 10° bacterial counts. In accordance with international guidelines, a 99.9% reduction in the bacterial count was considered indicative of bactericidal activity [22]. These findings confirmed the potency of propolis, which exhibited a bactericidal effect against both pathogens.

Against *L. monocytogenes*, propolis alone showed bactericidal activity at 125 mg/mL. In contrast, chestnut and thyme honeys, as well as thyme honey supplemented with propolis, did not reach the bactericidal threshold. However, chestnut honey enriched with propolis exhibited bactericidal activity at 500 mg/mL, indicating that the addition of propolis enhanced the effect, though not to the level of propolis alone.

For *C. perfringens*, propolis, chestnut honey, and chestnut honey + 10% propolis all showed bactericidal effects at 62.5 mg/mL. Thyme honey was the least effective, requiring 500 mg/mL to achieve the same effect. Nonetheless, its supplementation with propolis significantly improved its bactericidal activity, reducing the effective concentration to 250 mg/mL. This demonstrates that the addition of propolis enabled thyme honey to match the performance of the most active samples against this pathogen.

Overall, these results suggest that the bactericidal effect of propolis depends both on the matrix in which it is incorporated and on the target microorganism. These results highlight the progressive enhancement of antimicrobial efficacy observed with honey-propolis mixtures, emphasizing the potential role of phenolic compounds in bacterial growth inhibition. Given these findings (Figure 1 and Figure 2 and Table 2), chestnut honey was selected for further phenolic compound characterization, with a focus on its chemical profile before and after propolis addition. This approach aims to identify and quantify potential changes in key phenolic compounds that may contribute to the enhanced antimicrobial activity observed in chestnut honey + 10% propolis. Furthermore, propolis will serve as a reference active substance, enabling a direct comparison of its contribution to the overall bioactivity of the honey-propolis mixtures.

### 2.2. Bioactive Compound Characterization

Table 3 presents liquid chromatography–mass spectrometry–quadrupole time-of-flight (LC-MS-Q/TOF) data identifying phenolic compounds in propolis.

The phenolic profiles of chestnut honey, propolis, and their combination were analyzed using ultra-performance liquid chromatography coupled to photodiode array detection (UPLC-PDA), revealing key bioactive compounds responsible for antimicrobial activity. High concentrations of *p*-coumaric acid, pinobanksin-3-*O*-hexanoside, quercetin, and quercetin-3,7-dimethyl ether were identified in propolis (Table 4). The addition of 10% propolis to chestnut honey significantly increased the content of cinnamic acid derivatives, pinobanksin-3-*O*-hexanoside, sakuranetin, quercetin-3,7-dimethyl ether, and quercetin (Table 4). The presence of these bioactive components correlates with the enhanced antimicrobial efficacy observed in propolis-enriched formulations, as demonstrated in Figure 1 and Figure 2, which present the survival results.

## 3. Discussion

### 3.1. Antimicrobial Efficacy, Phenolic Compounds, and Comparison with Previous Studies

Several authors have reported the antibacterial activity of various honeys against *L. monocytogenes*. Inhibitory effects were observed for different Ukrainian honey types, *Helianthus*, *Robinia*, *Brassica*, or *Tilia* honeys, describing minimum inhibitory concentrations (MICs) from 94 to 188 mg/mL [23]. Russo et al. reported the inhibitory activity of Sicilian honeys—including chestnut and thyme—against *L. monocytogenes* using broth microdilution assays [24]. Interestingly, their results showed different percentages of inhibition for thyme and chestnut honeys, which the various geographical and botanical origins could explain [13]. Our study aligns with prior studies demonstrating the antibacterial effects of propolis (Italian origin) in dairy products and other food matrices against *L. monocytogenes* [25].

Our findings against *C. perfringens* are also in line with previous reports describing the antibacterial effects of various monofloral honeys, including acacia, citrus, sesame, cotton, and clover honeys from Egypt [26]. To the best of our knowledge, the antibacterial activities of chestnut and thyme honeys against *C. perfringens* have not been previously reported. However, the antibacterial effects of Chinese propolis extract have been shown against this foodborne pathogen [15].

Although no ethanol control was included in the present study, the residual ethanol concentration in the final test dilutions was below levels commonly associated with significant antibacterial activity. Several studies have demonstrated the antibacterial activity of ethanolic extracts of propolis (EEP) against *L. monocytogenes* and *C. perfringens*, attributing the effects primarily to the bioactive phenolic compounds in the propolis rather than to the ethanol solvent. For instance, a recent evaluation of Spanish propolis reported significant antimicrobial activity against *Listeria* strains using EEP, without ethanol-specific controls, and identified flavonoids and phenolic acids as the main contributors to the activity [27]. Similarly, the antimicrobial activity of Bulgarian propolis against anaerobic bacteria, including *Clostridium* species, was shown to exceed that of ethanol alone, supporting the role of phenolic constituents in the observed effects [28].

The enhanced activity observed in honey-propolis combinations highlights the role of propolis as a potent antimicrobial agent against *C. perfringens* and *L. monocytogenes* [15,25]. In contrast, Postali et al. reported no synergistic effect between honey and propolis from the Greek island of Samothrace against *L. monocytogenes* [17], underscoring the importance of composition variability in determining efficacy. However, we observed different effects on the antimicrobial activity between chestnut honey-propolis and thyme honey-propolis combinations, with chestnut honey being the most effective. A study comparing different Spanish honeys, including chestnut and thyme, showed that chestnut honey had greater antioxidant potential and antibacterial activity, which could be explained by the difference in the profile of phenolic compounds associated with the different botanical origins [29]. León-Ruiz et al. reported that the floral origin of Spanish honeys affects their chemical composition and functional properties, chestnut honey had the highest total phenolic content, and thyme honey showed the highest vitamin C level. Moreover, only chestnut honey showed antimicrobial activity against *E. coli* and *S. aureus* [11].

The increased antimicrobial activity observed in our study is supported, at least in large part, to the chemical composition of the propolis extract, characterized by the high content of polyphenols, flavonoids, and aromatic acids in propolis [30,31]. Compounds such as caffeic acid, ferulic acid, *p*-coumaric acid, chrysin, sakuranetin, pinocembrin, pinobanksin derivatives, quercetin, and acacetin have previously been identified in Spanish propolis and linked to antibacterial properties [30,31]. In chestnut honey, Combarros-Fuertes et al. reported the presence of ellagic acid, quercetin, pinobanksin, kaempferol, and chrysin [29]. Our results confirm and expand upon these findings, highlighting the importance of *p*-coumaric acid, pinobanksin-3-*O*-hexoside, and quercetin derivatives as key contributors to the observed antimicrobial activity.

For instance, caffeic acid phenethyl ester, pinobanksin, and pinocembrin have been reported to be active against *C. perfringens* [15], showing MICs of 12.5, 50, and 50 µg/mL, respectively, and minimum bactericidal concentrations (MBCs) of 75, 100, and 100 µg/mL. Pernin et al. evaluated the antibacterial activities of *p*-coumaric, ferulic, and caffeic acids against *L. monocytogenes*, reporting MICs of 18.03 mmol/L for caffeic acid, 13.60 mmol/L for ferulic acid, and 15.44 mmol/L for *p*-coumaric acid, which correspond to approximately 3.25, 2.64, and 2.53 mg/mL, respectively [32]. In our study, *p*-coumaric acid and pinobanksin-3-*O*-hexanoside were detected in chestnut honey, propolis, and chestnut honey + 10% propolis at concentrations higher than those reported in these previous studies. The antimicrobial activity of the samples cannot be attributed solely to individual phenolic compounds but may result from synergistic interactions among multiple bioactive compounds.

The synergy between honey and propolis in bacterial inhibition may stem from complementary mechanisms of action, such as membrane disruption, interference with bacterial enzyme systems, and the modulation of metabolic or quorum-sensing pathways [15,33,34,35] Additionally, certain phenolics have been implicated in the disruption of biofilm formation and interference with bacterial communication (quorum sensing) [33], which may further explain the enhanced efficacy observed in honey-propolis mixtures. Specifically, against *L. monocytogenes*, caffeic, *p*-coumaric, and ferulic acids exert antimicrobial effects mainly through their undissociated form, which are responsible for inhibiting bacterial growth [35]. Chinese propolis extract caused morphological elongation, bacterial cell wall damage, and intracellular material leakage in *C. perfringens*, leading to alterations in metabolism, which may be attributed to phenolic compounds [15].

The chemical analysis revealed that the addition of 10% propolis to chestnut honey significantly increased the concentration of antimicrobial phenolic compounds, including cinnamic acid derivatives, pinobanksin-3-*O*-hexoside, sakuranetin, quercetin-3,7-dimethyl ether, and quercetin. These compositional changes were consistent with the enhanced bactericidal activity seen in the biological assays, indicating that these compounds could serve as chemical markers of bioactivity.

### 3.2. Future Directions, Limitations, and Potential Applications

Foodborne pathogens continue to represent a major public health concern. According to the WHO, foodborne diseases account for 33 million years of healthy life lost annually, despite being largely preventable [36]. The target pathogens of this study—*L. monocytogenes* and *C. perfringens*—are responsible for serious foodborne illnesses with wide-reaching clinical and economic impacts. They are not only frequently implicated in foodborne outbreaks but also have broader clinical implications beyond the gastrointestinal tract. To better understand the relevance of the observed antimicrobial effects, it is essential to consider the microbiological and clinical characteristics of these pathogens.

*L. monocytogenes* is a Gram-positive and non-spore-forming bacterium [37]. This microorganism causes serious animal and human diseases and is linked to a mortality rate of 20–30% worldwide [37,38]. Consequently, *L. monocytogenes* significantly affects the food safety of milk and dairy products, vegetables, meat, poultry, and seafood products [37,39]. After consumption of contaminated food, this microorganism can cross the intestinal barrier and enter the bloodstream, and it can also traverse the blood–brain or the fetoplacental barrier and cause meningitis, sepsis, or abortion [39].

*C. perfringens* is a spore-forming bacterium and is associated with veterinary and human diseases [40]. This Gram-positive and anaerobic bacterium can cause illness through the consumption of contaminated food and is the fourth most frequent agent of food-borne outbreaks in Europe [41]. *C. perfringens* is usually found in meat products and chicken or vegetables and crops [41]. An important fact is that different strains of *C. perfringens* secrete more than 20 toxins or enzymes, which could be involved in the pathophysiology [42]. In addition, other virulence factors of this microorganism are its survival in aerobic environments, the production of toxic gases, and accelerated growth [42].

According to the Centers for Disease Control and Prevention (CDC), *C. perfringens* causes nearly 1 million foodborne illnesses in the United States annually, and *Campylobacter jejuni* is the most common pathogen, followed by *Listeria* [43,44]. The antimicrobial activity of the studied samples, particularly propolis and honey-propolis mixtures, suggests promising applications in food preservation and as nutraceutical ingredients. Although propolis alone showed the most potent bactericidal effect, its intense taste and aroma may limit its sensory acceptance in food products [35]. In contrast, honey-propolis mixtures may offer a better balance between efficacy and consumer acceptability. This was confirmed in a recent study from our group, in which honey-propolis formulations were applied to reduced-nitrite dry fermented sausages. The results demonstrated not only microbiological stability but also acceptable sensory properties, indicating that the strong sensory profile of propolis can be successfully balanced through appropriate formulation [21]. This supports the feasibility of their application in real food systems. In pharmaceutical applications, various strategies such as encapsulation have been proposed to reduce the strong and sometimes unpleasant taste of propolis [45]. Our results suggest that combining propolis with other bee products, particularly honey, may offer a simpler and effective alternative to improve sensory acceptability while maintaining or even enhancing antimicrobial efficacy.

*L. monocytogenes* and *C. perfringens* strains were evaluated, and although they are highly relevant foodborne pathogens, a broader spectrum of microorganisms—including antibiotic-resistant strains—should be included in future investigations. On the other hand, while in vitro assays provide important initial insights, they do not fully represent the complexity of food matrices or physiological environments. Therefore, in vivo studies and applications in real food systems are essential to confirm the antimicrobial potential and practical feasibility of these formulations. The combination of honey and propolis may exert antibacterial effects through multiple mechanisms. Propolis has been shown to affect microbial cell membrane permeability, disrupt membrane potential, and inhibit ATP production [46], while honey’s antimicrobial properties are attributed to factors such as low pH, high sugar content, and the presence of hydrogen peroxide and phenolic compounds [47]. Given these diverse mechanisms, future studies should investigate the specific interactions between honey and propolis combinations to elucidate their synergistic effects and potential applications in antimicrobial therapies.

Importantly, the antimicrobial patterns observed in vitro are consistent with previous in situ findings from our group, where chestnut honey-propolis mixtures were applied to nitrate-reduced fermented meat products, and no pathogens were detected after ripening [21]. However, the previous study did not isolate the specific contribution of these mixtures to microbial inhibition, nor were direct antimicrobial assays conducted. The present work provides the first controlled experimental evidence of their antibacterial effects, supporting their role as functional antimicrobial ingredients.

These findings contribute to the growing body of evidence supporting natural antimicrobial strategies and reinforce the potential of using targeted combinations of honey and propolis, selected based on their botanical and geographical origins, to enhance antimicrobial activity against specific foodborne pathogens. This tailored approach could support the formulation of bioactive ingredients aimed at mitigating contamination risks in vulnerable food matrices. Moreover, it may extend beyond food preservation to pharmaceutical applications, particularly in the prevention or treatment of gastrointestinal infections and topical wound management.

## 4. Materials and Methods

### 4.1. Chemicals and Reagents

Agar and Brain Heart Infusion (BHI) were purchased from Becton Dickinson (Franklin Lakes, NJ, USA). Formic acid and acetonitrile were obtained from Sigma-Aldrich (St. Louis, MO, USA). Standards such as *p*-coumaric acid and quercetin were purchased from Extrasynthese (Lyon, France). The water used in the experiments was purified by a Milli-Q system (Merck Millipore, Burlington, MA, USA).

### 4.2. Raw Materials

**Figure 3:**
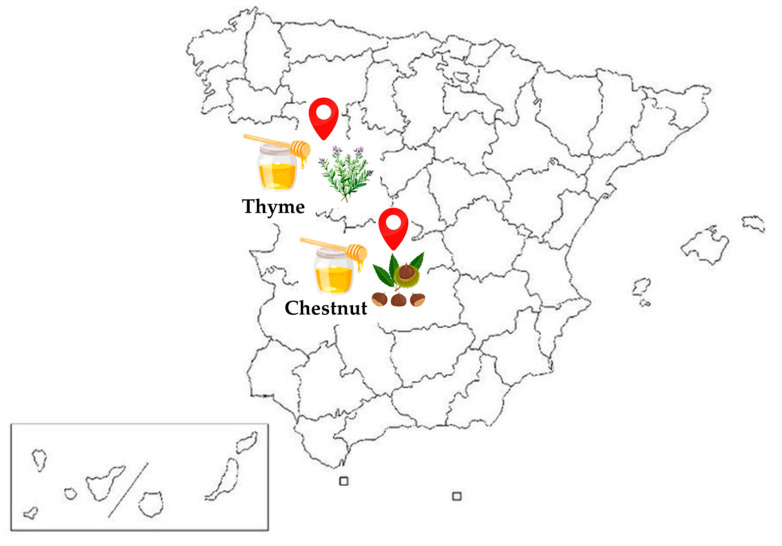
Provinces of origin of the honey samples in Spain. Zamora (west): thyme honey; Toledo (central area): chestnut honey.

Thyme and chestnut honey samples were obtained from beekeepers in two Spanish regions (Figure 3), in January 2021.

Table 5 shows the classification, family, and scientific name of the plants that form the principal flora of the honey samples, as well as the geographic region of the honey and propolis samples. The values of the physicochemical parameters of both honeys were within the established limits by the Spanish Royal Decree-Law (RD) 1049/2003 [48].

Thyme and chestnut honey were mixed with propolis (10%; *w*/*w*) to obtain the mixtures evaluated in this study. The 10% propolis concentration used in the present work reflects the same formulation previously tested in nitrate-reduced fermented meat products, where it demonstrated in situ microbiological efficacy under real conditions [21].

All samples were obtained as previously described by Sánchez-Martín et al. [48]. Briefly, the samples were weighed and diluted in distilled water to a final concentration of 1 g/mL. Stocks of samples were filtered (0.45 µm Minisart^®^, Sartorius, Goettingen, Germany) and stored at −20 °C.

### 4.3. Foodborne Pathogen Strains and Culture Conditions

The antibacterial activity of bee products was assessed against *Listeria monocytogenes* CECT 4032 and *Clostridium perfringens* CECT 376, used as reference strains, obtained from the Spanish Type Culture Collection (CECT, Valencia, Spain). The strains were stored at −80 °C in BHI broth supplemented with 25% (*v*/*v*) glycerol until use. Strains were reactivated from frozen stocks in Brain Heart Infusion broth at 37 °C for 24 h. For *C. perfringens*, anaerobic conditions were maintained using the anaerobic cabinet (BACTRON ™ Anaerobic/Environmental Chamber, patented by Anaerobe Systems. Biogen, Madrid, Spain) and the anaerobic jar (VWR International, Radnor, PA, USA).

### 4.4. Evaluation of the Antibacterial Activity of Bee Products

The antibacterial activities of thyme honey, chestnut honey, and propolis, as well as their combined formulations (thyme honey + 10% propolis and chestnut honey + 10% propolis), were evaluated against *L. monocytogenes* and *C. perfringens* using a broth microdilution assay in 96-well microplates. According to del Castillo et al. [49], reactivated bacterial cultures were inoculated into BHI (10^5^ CFU/mL) and mixed with serial dilutions of the bee products (1/2 to 1/256, prepared from 1 g/mL stock solutions). Plates were incubated at 37 °C for 20 h (under anaerobic conditions for *C. perfringens*), and absorbance at 600 nm was recorded at the beginning and end of the assay using a CYTATION 5 microplate spectrophotometer (BioTek Instruments, Winooski, VT, USA). A significant inhibitory concentration was defined as the dilution that resulted in a statistically significant reduction (*p* < 0.05) in bacterial growth (final absorbance) compared to an untreated control.

Subsequently, 10 µL of inoculum from each well of 96-well plates were plated on BHI agar plates and incubated for another 24 h at 37 °C. Colony-forming unit (CFU) counts were conducted in a Stuart colony counter SC6 (VWR International, Radnor, PA, USA), and the number of CFU/mL was obtained.

All experiments were conducted using two independent biological replicates (separate bacterial cultures and freshly prepared bee product solutions), each analyzed in duplicate to account for technical variability. This design, commonly accepted in microbiological studies involving broth microdilution assays, ensures reproducibility while optimizing the use of biological materials. In addition, two complementary techniques were employed—optical density measurement (OD600) and viable cell counts (CFU/mL)—to robustly assess bacterial growth inhibition and validate the results across methodologies. The low variability observed between replicates confirmed the stability and reliability of the assay. This approach is aligned with published protocols [49] and ensures that statistically significant effects reflect true biological responses rather than technical noise.

### 4.5. Analysis of Individual Polyphenols

The samples (chestnut honey, chestnut honey + 10% propolis, and propolis) were filtered through a Hydrophilic PTFE 0.20 µm (Millex Samplicity Filter, Merck, Darmstadt, Germany). Qualitative (LC-MS-Q/TOF) and quantitative (UPLC-PDA) analyses of polyphenols (flavonols at 360 nm and phenolic acids at 320 nm) were carried out following the method described by Wojdyło et al. [50]. The separation of individual compounds was carried out by using an ACQUITY UPLC BEH C18 column (1.7 µm, 2.1 × 100 mm, Waters Corporation, Milford, USA) at 30 °C. The injection of 5 µL samples and elution was completed in 15 min using a sequence of linear gradients and isocratic flow rates of 0.42 mL/min. The mobile phase comprised solvent A (2.0% formic acid, *v*/*v*) and solvent B (100% acetonitrile). The program initiated with gradient elution from 99% to 65% solvent A (0–12 min), followed by reducing solvent A to 0% for column conditioning (12.5–13.5 min) and returning the gradient to the initial composition (99% A) until 15 min, where it was held constant to re-equilibrate the column. The identification of polyphenols was carried out using a QTof mass spectrometer (Waters Corp.; Milford, MA, USA) equipped with an ESI source, operating in negative mode, on the basis of fragmentation patterns and PDA profiles. Mass spectrometer source parameters were as follows: capillary (3 kV), sampling cone (18), extraction cone (4), temperature (120 °C), desolvation temperature (350 °C), cone gas (50 L nitrogen/h), and desolvation gas (600 L nitrogen/h).

The calibration curves were made for the standard *p*-coumaric acid (*r*^2^ = 0.9835) and quercetin (*r*^2^ = 0.9889) at concentrations ranging from 0.05 to 0.5 mg/mL. The identified compounds include the following: sakuranetin, quercetin-3,7-dimethyl ether, quercetin-3-methyl ether, and pinobanksin-3-*O*-hexanoside, calculated using quercetin as a reference compound. The remaining identified compounds were calculated using *p*-coumaric acid as a reference compound. All measurements were replicated three times, and the results were expressed as mg per mL (as the liquid form of the sample).

### 4.6. Statistical Analysis

All data are expressed as the mean ± standard deviation (SD) from two independent experiments for the antibacterial activity assays (two independent biological replicates were performed, each consisting of two technical replicates, *n* = 2) and three independent experiments for the analysis of individual polyphenols (*n* = 3). One-way ANOVA followed by Tukey’s post hoc test was used to compare differences among multiple treatments. When comparing two specific groups, the Student’s *t*-test was applied. A *p*-value < 0.05 was considered statistically significant. Statistical analyses were performed using Statgraphics Centurion 19 Software (Statgraphics Technologies, Inc., The Plains, VA, USA).

## 5. Conclusions

This study demonstrates that the antimicrobial potential of honey is significantly enhanced by the addition of propolis, particularly against *Clostridium perfringens* and *Listeria monocytogenes*. The observed bioactivity appears to be linked to the presence of key phenolic compounds such as p-coumaric acid, pinobanksin-3-O-hexoside, and quercetin derivatives, whose synergistic interactions may contribute to the improved efficacy of these mixtures.

Future research should explore the molecular mechanisms underlying this enhanced activity, including effects on bacterial membrane integrity, metabolism, and quorum-sensing pathways. Evaluating the effectiveness of these combinations in real food systems will also be essential to assess microbial stability. In vivo infection models are needed to further investigate safety, bioavailability, and therapeutic potential in clinical or veterinary applications.

From a formulation standpoint, systematic characterization of the phenolic profiles of honey and propolis from diverse botanical and geographical origins would enable the development of customized combinations targeted to specific pathogens or application contexts. Such formulations could serve as natural preservatives in food systems or as adjuncts to antimicrobial therapy, paving the way for multifunctional nutraceuticals and pharmaceutical-grade bioactive ingredients.

In addition to their antimicrobial effects, honey and propolis are increasingly investigated in galenic preparations such as gels, ointments, and hydrogel-based wound dressings due to their wound-healing, antioxidant, and anti-inflammatory properties. Their incorporation into natural health products tailored to a wide range of populations opens promising avenues for the development of multifunctional bioactive agents. Their potential use in treating ulcers, burns, or chronic wounds, where localized antimicrobial action and tissue regeneration are essential, warrants further investigation.

Altogether, our findings support the rational development of honey-propolis mixtures as dual-purpose antimicrobial formulations. The present work provides the first direct evidence of their pathogen-specific action and validates their relevance in both food preservation and pharmaceutical contexts. This dual functionality, combining antimicrobial efficacy with antioxidant and sensory advantages, positions honey-propolis mixtures as promising candidates for clean-label preservatives, nutraceuticals, and functional pharmaceutical ingredients aimed at controlling foodborne and clinical infections.

## Figures and Tables

**Table 1:** Colony-forming unit (CFU) counts for *L. monocytogenes* and *C. perfringens* following treatment with bee product samples.

Foodborne Pathogen	Concentration (mg/mL)	Thyme Honey + 10% Propolis	Chestnut Honey + 10% Propolis	Propolis
*L. monocytogenes*	15.6	>10^7^	>10^7^	>10^7^
	31.2	>10^7^	>10^7^	>10^7^
	62.5	>10^7^	>10^7^	>10^7^
	125	<10^3^	<10^3^	<10^3^
	250	<10^3^	<10^3^	<10^3^
	500	<10^3^	<10^3^	<10^3^
*C. perfringens*	15.6	>10^7^	>10^7^	<10^3^
	31.2	<10^3^	<10^3^	<10^3^
	62.5	<10^3^	<10^3^	<10^3^
	125	<10^3^	<10^3^	<10^3^
	250	<10^3^	<10^3^	<10^3^
	500	<10^3^	<10^3^	<10^3^

**Table 2:** Sample concentrations (mg/mL) that decreased bacterial growth by 99.9% (bactericidal effect).

Foodborne Pathogen	Thyme Honey	Thyme Honey + 10% Propolis	Chestnut Honey	Chestnut Honey + 10% Propolis	Propolis
*L. monocytogenes*	>500	>500	>500	500	125 *
*C. perfringens*	500 ^c^	250 ^b^	62.5 ^a^	62.5 ^a^	62.5 ^a^

*L. monocytogenes*: asterisk indicates a significant difference between chestnut honey + 10% propolis and propolis (*p* < 0.05; t-Student test). *C. perfringens*: different letters differ significantly (*p* < 0.05; Tukey’s HSD test).

**Table 3:** Phenolic compounds present in propolis, detected using LC-MS-Q/TOF.

R_t_	nm	MS	MS/MS	Name
4.64	309	163.03	145.02/119.05	*p*-coumaric acid
5.91	320	411.99	329.07/179.03/135.04	cinnamic acid derivatives
6.58	322	369.11	271.06/253.04/197.05	pinobanksin-3-*O*-hexanoside
6.98	323	285.04	165.01	sakuranetin
8.88	321	329.13	317.06/207.06/179.03	quercetin-3,7-dimethyl ether
9.94	254, 368	285.04/301.03		quercetin
12.98	323	253.05	178.53/134.03	caffeoyl glycerol
10.48	355	315.05		quercetin-3-methyl ether
12.82	305	283.06		acacetin

R_t_—retention time [min].

**Table 4:** Content of bioactive compounds in chestnut honey, propolis, and chestnut honey + 10% propolis (mg/mL), quantified by UPLC-PDA.

Bioactive Compound	Chestnut Honey	Chestnut Honey + 10% Propolis	Propolis
*p*-coumaric acid	63.59 ± 3.46 ^b^	49.96 ± 0.13 ^ab^	549.02 ± 17.89 ^c^
cinnamic acid derivatives	ND	106.60 ± 5.78 ^a^	172.79 ± 11.88 ^b^
pinobanksin-3-*O*-hexanoside	2.61 ± 0.03 ^a^	49.42 ± 0.20 ^b^	457.31 ± 14.65 ^c^
sakuranetin	2.26 ± 0.01 ^a^	6.55 ± 0.03 ^b^	79.48 ± 0.33 ^d^
quercetin-3,7-dimethyl ether	ND	96.57 ± 0.65 ^a^	761.46 ± 26.30 ^b^
quercetin	3.20 ± 0.01 ^a^	118.71 ± 5.09 ^c^	755.35 ± 3.65 ^d^
caffeoyl glycerol	ND	ND	194.28 ± 10.41 ^b^
quercetin-3-methyl ether	21.12 ± 0.71 ^b^	ND	7.34 ± 0.04 ^a^
acacetin	227.79 ± 13.58 ^b^	ND	74.89 ± 0.79 ^a^

ND: not detected. Data are expressed as the mean ± SD (*n* = 3). The values in the rows followed by different letters indicated significant statistical differences (*p* < 0.05; Tukey’s HSD test).

**Table 5:** Honey and propolis samples.

Sample	Scientific Name	Classification	Family	Geographic Region
Thyme honey	*Thymus* spp.	Monofloral	*Lamiaceae*	Spain, Zamora
Chestnut honey	*Castanea sativa*	Monofloral	*Fagaceae*	Spain, Toledo
Propolis tincture *	-	-	-	Spain, Zamora

* Propolis extract dissolved in 70% organic ethanol.

## Data Availability

The raw data supporting the conclusions of this article will be made available by the authors on request.

## Abbreviations

The following abbreviations are used in this manuscript:

BHI	Brain heart infusion
CDC	Centers for Disease Control and Prevention
CFUs	Colony-forming units
*C. perfringens*	*Clostridium perfringens*
ECDC	European Centre for Disease Prevention and Control
*E. coli*	*Escherichia coli*
EFSA	European Food Safety Authority
LC-MS-Q/TOF	Liquid chromatography–mass spectrometry–quadrupole time of flight
*L. monocytogenes*	*Listeria monocytogenes*
MBC	Minimum bactericidal concentration
MIC	Minimum inhibitory concentration
SD	Standard deviation
*S. aureus*	*Staphylococcus aureus*
UPLC-PDA	Ultra-performance liquid chromatography coupled to photodiode array detection
WHO	World Health Organization
